# Using image augmentation techniques and convolutional neural networks to identify insect infestations on tomatoes

**DOI:** 10.1016/j.heliyon.2024.e41480

**Published:** 2024-12-26

**Authors:** Moy'awiah Al-Shannaq, Shahed N. Alkhateeb, Mohammad Wedyan

**Affiliations:** Faculty of Information Technology and Computer Sciences, Yarmouk University, Jordan

**Keywords:** Agriculture, Crop disease, New technologies, Augmented reality, Deep learning, Convolutional neural networks

## Abstract

Pest insects are a danger to both regional and global food security. In Jordan, the most productive crop is tomato. Jordan's agriculture output is threatened by insect infestations. The study intends to use a deep learning model called convolutional neural networks on a dataset that includes eight categories of insect pest images. A dataset was used and a group of images from reliable sources were added to it. The image collection was analyzed, and an image augmentation technique was used to increase the number of images, which reached 5894 after image augmentation. The data was split among 80 % training and 20 % validation. Convolutional Neural Networks trained on the data achieved 90 % training accuracy, 85 % testing accuracy, and 87 % validation accuracy. A high-accuracy deep learning model was developed that may be utilized on mobile applications to detect pests that affected crops to assist farmers. The original database used was small in size. When tested on deep learning and machine learning systems, the accuracy was very low, reaching 50–60 % without image augmentation, despite image enhancement techniques.

## 1 Introduction

In recent years, agriculture in Jordan has witnessed significant modernization due to the need to improve food security levels in Jordan. Agricultural challenges have led to a sharp increase in the use of agricultural technology. One of the major economic contributors to Jordan is the agricultural industry [1]. The agricultural sector in Jordan, like any country in the world, faces more issues due to product insects that harm cultivated plants. The major challenges faced by those interested in agriculture are pest and disease attacks that reduce crop productivity. However, it is possible to control pest attacks at the early attack stage to reduce the use of pesticides and maintain early crop vitality. Mobile applications [2] capable of providing accurate identification of the disease that has affected the crop instead of manual detection. The importance of having a system that helps in early detection of agricultural insects that infect various crops lies in increasing the rate of plant products and achieving high returns without any additional cost to the farmer.

The farming sector has faced transformative alteration in evolving from Agriculture 2.0 to Agriculture 5.0. Agriculture 5.0 act as revolutionary stage, emerging new technologies to improve efficiency that adapts to the needs of fields and livestock. For instance, the integration of robotics and augmented reality represents a major leap in agricultural tech-nology, enabling real-time monitoring and automation in agricultural practices. Artificial Intelligence (AI) and big data are key components of Agriculture 5.0, providing insights for predictive analytics and decision-making [3]. Smart precision agriculture can be enhanced with the help of the latest Internet of Things (IoT), unmanned aerial vehicles (UAV), aug-mented reality (AR) systems, and machine learning (ML) algorithms [4].

The growing interest in new technologies, such as virtual reality (VR), has led researchers to change how user studies are conducted in the field human-robot interaction (HRI). It has shown that evaluating prototyping solutions in VR will provide multiple benefits in terms of cost and safety. VR is widely used in various fields, such as medicine, education, industry, aerospace, architecture, history, and agriculture. Immersive virtual reality allows the user to “be part of the environment” by being exposed to a digitally created world [5]. VR technology has the possibility to guarantee that agricultural information reaches audiences in a significative way, serving as a complement or alternative to traditional media. VR can create a robust link with the audience through interactive and immersive virtual experiences that provide verifiable cases to form opinions [6].

One of the challenges facing developing countries is the lack of a sufficiently trained workforce [7]. Smart farming is facilitated through Augmented Reality (AR), which also helps in monitoring crop health. AR can help new farmers feel comfortable when using agricultural machinery [8]. When the user opens the augmented reality app, they will see a real video captured by the camera. At the same time, virtual graphics and relevant text information can be seen overlaid on relevant objects, making the real world richer and more diverse. Improving the interaction between people and the real environment, and the development of mobile terminals such as smartphones provide the necessary conditions for creating reality applications [9]. Virtual and augmented reality, mobile applications, and games have enhanced agricultural education and learning experiences [10].

In the field of vocational education, vocational colleges, and training institutions can effectively enhance students’ learning and improve their learning efficiency through the use of VR technology, which solves problems such as high teaching expenses and spatial limitations inherent in practical training [11]. The contribution lies in dealing with a small data sample and augmenting these images to achieve very acceptable results. Also, the original database used was small in size. When tested on deep learning and machine learning systems, the accuracy was very low, reaching 50–60 %, despite image enhancement techniques.

## 2 Related work

In this section, a collection of research related to the subject of study, which is AR and VR in different fields.

In the study [12] when there are not enough training examples, data augmentation is crucial. To train an industrial steel surface defect classification network, the performance of the network is mostly dependent on the availability of high-quality training samples. This study focuses on enhancing data augmentation for this purpose.

Finding an adequate dataset for this application in real-world scenarios is exceedingly chal-lenging. This study presents a unique offline pre-augmentation network (PreAugNet) that functions as a class boundary classifier to enhance imagined augmentation and efficiently screen the quality of the enhanced samples. PreAugNet considerably improves accuracy by 3.3 % (AOI dataset) and 6.25 % (MT dataset).

Furthermore, in the study [13] to detect infections in olive crops, machine learning and deep learning techniques were used. Multiple sources of image data were combined into a single dataset. The data was divided into 30 % for testing and 70 % for training. For features extraction, SqueezeNet deep learning techniques were applied. Both Support Vector Machine and Artificial Neural 10.13039/100031212Network models were used for data training. Results of the study showed that the Artificial Neural 10.13039/100031212Network outperformed the Support Vector Machines model, which had a score of 85.5 %, with 92.9 % accuracy. The study found that images of olive illnesses were sufficiently apparent that image augmentation methods were not required. In a study conducted in 2024 [14], the factors affecting farmers' intention to use smart glasses and AR technologies were studied and analyzed. The results showed that the per-ceived benefit of adopting new technologies directly influenced agricultural stakeholders’ acceptance of smart glasses as a new and innovative tool to support on-farm activities [15]. As well, Researchers used AR, an innovative technology that allows digital data to be blended in real time with the environment in which the user lives. AR used to monitor and evaluate cows for producers. In essence, AR provides farmers with real-time data on the health and well-being of their animals, allowing their livestock to be managed more efficiently and effectively. In addition, AR improves the accuracy of estimating milk production and better identify potential health issues.

Researchers [6] developed a virtual farm simulation to provide realistic on-farm experiences raising cows. Users are able to explore locations where dairy cows are raised within the virtual farm and gain insight into dairy production processes using the head-mounted display. This simulation was shown at local libraries, with an audience of 48 participating. The study underscores the potential of extended reality (XR) technologies to address challenges in the agricultural sector, emphasizing the need for further research to improve interactions and overcome health and privacy concerns.

In 2023, a study conducted by Ref. [9] presented a model combining using of feature gradient graphs (HOG), support vector machines (SVM), and augmented reality. (HOG-SVM Recognition Model) For plant disease recognition, HOG- SVM obtained faster training results and higher accuracy recognition results than deep models such as YOLOv3, SSD 512, and Fast R-CNN. HOG-SVM has an average accuracy of 77 %.

In 2022, a study by Ref. [16] proposed four Convolutional Neural Network (CNN) models: Xception, MobileNet, MobileNetV2, and EfficientNetB7. In identifying lesions using deep learning and AR, the EfficientNetB7 approach achieved a classification accuracy of 95.51 percent, Xception obtained a classification accuracy of 94.3 percent, and MobileNet obtained a classification accuracy of 94.5 percent. In their study [16] as well, AR was integrated on a mobile device in order to detect agricultural pests, take control measures, and monitor plant diseases in real-time. This was achieved by using smartphone camera to capture images of pests through a mobile app built using the Flutter framework. The images are then analyzed in the app using different transfer learning-based models of the available Kaggle dataset to identify lesions. Additionally, a study carried out by Ref. [8] proposed leaf disease classification by developing CNN models for semantic segmentation and classification of agricultural pests using a yolov5 object detector, the real-time object detection accuracy range is 70%–90 %.

In [17] study, the researchers developed a research-based theoretical and practical frame-work to help teachers, evaluators, and researchers further examine the qualitative and quantitative data of students engaged in virtual reality. Whereby virtual reality, when appropriately integrated with VRFRAM, improves students’ agricultural knowledge by increasing their awareness and engagement in the classroom.

Moreover, an image processing technique was proposed by Ref. [2], for this technique images of lesions are taken and subjected to various pre-processing processes to reduce noise and improve the images. The user can identify the infection stage of cultivated plants by applying a classification algorithm using the CNN model. CNN identifies the lesion type with up to 90 % accuracy. A study by Ref. [18] proposed a prototype of augmented reality-based mobile applications to detect crop diseases for farmers.

In India, farmers generally do not have formal training in pest identification and treat-ment. Insects of all kinds are treated equally without giving any importance to the fact that some of them can be good. Accordingly, study conducted by Ref. [19] proposed detecting crop-infecting insects by developing a mobile application based on augmented reality. Table 1 contains reviews of research on the application of AR and VR technology in agriculture.

**Table 1:** A review of prior research on the use of AR and VR technology in agriculture to diagnose plant diseases.

Ref.	Year	Topic	Methodology	Dataset	Result(Accuracy)
[5]	2024	VR in grape vineyards	Natural Language Under-standing module	Italian speech dataset from 81 participants	Immersive Virtual Environment (IVE) has a high sense of presence = 4.36/5 77 %
[9]	2023	AR-Vegetable disease	AR-assisted HOG-SVM-based mobile	Vegetable diseases database (Five dis-eases)	
[8]	2022	Leaf's Dis-eases Using AR	CNN with yolov5	COCO dataset	70%–90 %
[16]	2022	Pest Identification and Control with AR.	Xception, MobileNet, Mo-bilenetV2 and EfficientNetB7	Kaggle dataset	EfficientNetB7 95.15 %
[2]	2021	Identify the stage of the damaged plant	CNN	IP102_V1.1 dataset for whiteflies	90 %

## 3 Methodology

The study's working dataset and techniques are described in detail and rendered clearly in this part.

### 3.1 Overall methodology

**Fig. 1:**
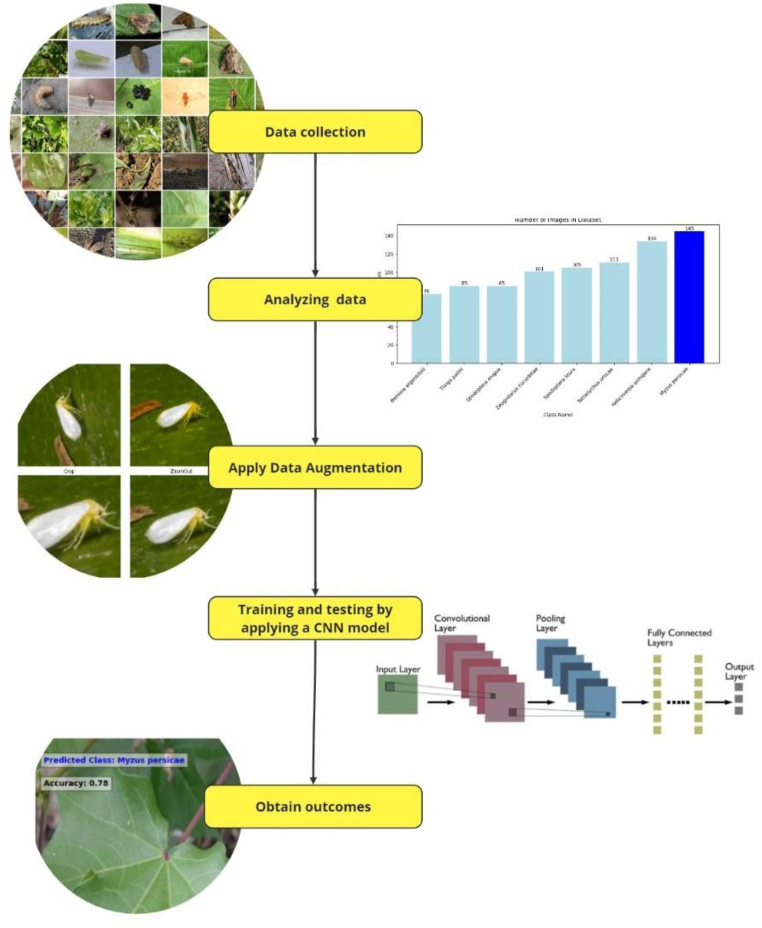
Overall methodology.

The research approach employed in this study is depicted in Fig. 1. 1) A collection of images of insect pests that endanger and infect crops was taken from an image dataset [20]. A website specializing in pest illnesses [21] on crops was searched to manually enhance the number of images in the basic dataset. 2) After analyzing the image data, the sizes of the images were standardized to (224 × 224 3). Data augmentation was used to a collection of images to expand the amount of data utilized and enhance the model's learning accuracy.

4) Using a deep learning CNN model to train and test the data after splitting the data into training and validation sets. 5) Record the accuracy outcomes.

### 3.2 Dataset description

As per the Department of Statistics of Jordan, tomatoes are the most planted vegetable in 2022 [22]. Consequently, research has been done to use deep learning algorithms to detect insect pests that infect tomatoes [20]. To integrate it into an AI model to quickly and effectively detect insect pests, a database of tomato illnesses was compiled from many trustworthy sources [21].

Eight categories of insect disease groups are included in the database: “Myzus persicae”, “Zeugodacus cucurbitae”, “Thrips palmi”, “Tetranychus urticae”, “Spodoptera litura”, “Bemisia argentifolii”, “Helicoverpa armigera”, and “Spodoptera exigua".

**Fig. 2:**
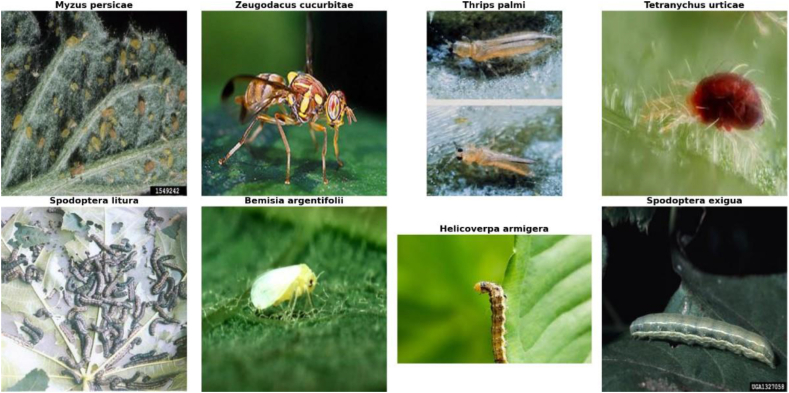
A group of insect pests that affect tomatoes used in the research [20,21].

Fig. 2 shows a set of images from the image database that were used and collected before image processing.

**Table:** 

Pseudocode Methodology
1. Import deep learning libraries (TensorFlow, OpenCV, and PIL). 2. Load and preprocess the dataset - Load training and validation datasets - Split the dataset into training and validation sets 3. Apply an image augmentation techniques. - Specify augmentations (e.g., rotation, flipping, zooming, brightness adjustments) 4. Apply augmentations to the training dataset. 5. Build the CNN model - Define the architecture of the CNN - Specify the input shape to match the augmented data dimensions 6. Train the model - Use the augmented training data. - Monitor validation performance for early stopping or tuning. 7. Evaluate the model - Test the model. - Compare performance metrics with and without augmentation. 8. Save and visualize results. - Save the trained model. - Plot training and validation accuracy.

**Fig. 3:**
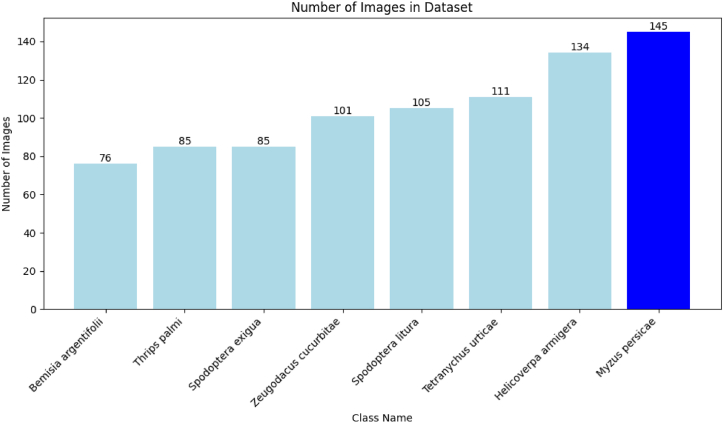
The number of images used in the search is arranged from smallest to largest.

In Fig. 3, and Table 2 the number of images of the pests used in the research is shown, arranged from smallest to largest, the smallest type used is the Bemisia argentifolii, where the number of images in this category reached 76 images, and the largest number of images is in the Myzus persicae category, where the number of images reached 145.

**Table 2:** Dataset description.

Class	The number of images used	The number of images used
	before data augmentation	after data augmentation
Myzus persicae	145	1015
Zeugodacus cucurbitae	101	707
Thrips palmi	85	595
Tetranychus urticae	111	777
Spodoptera litura	85	595
Bemisia argentifolii	76	532
Helicoverpa armigera	134	938
Spodoptera exigua	105	735
Results	%60	%85

#### 3.2.1 Standardizing the size of images and using image augmentation

Deep learning is a tool with very valuable implications. It extracts features from raw data to save time and effort for many applications. A deep learning model is capable of learning and extracting features from raw data by itself without any external intervention. Moreover, feature extraction techniques with shallow learning rely on the user's expertise to choose a robust feature extraction algorithm [23].

**Fig. 4:**
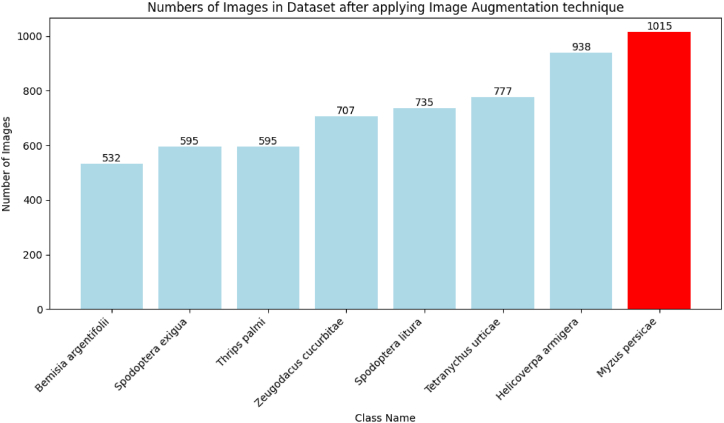
Increasing the number of images after applying image augmentation techniques.

Applying the standardization of image dimensions (length ∗ width) to 224 ∗ 224 was done in this phase. The number of samples in the original dataset was increased by using image augmentation techniques [24], which included flipping, cropping, zooming out, brightness, rotation, saturation, and grayscale augmentation. Fig. 4 shows the increase in the number of images after using an image augmentation technique.

**Fig. 5:**
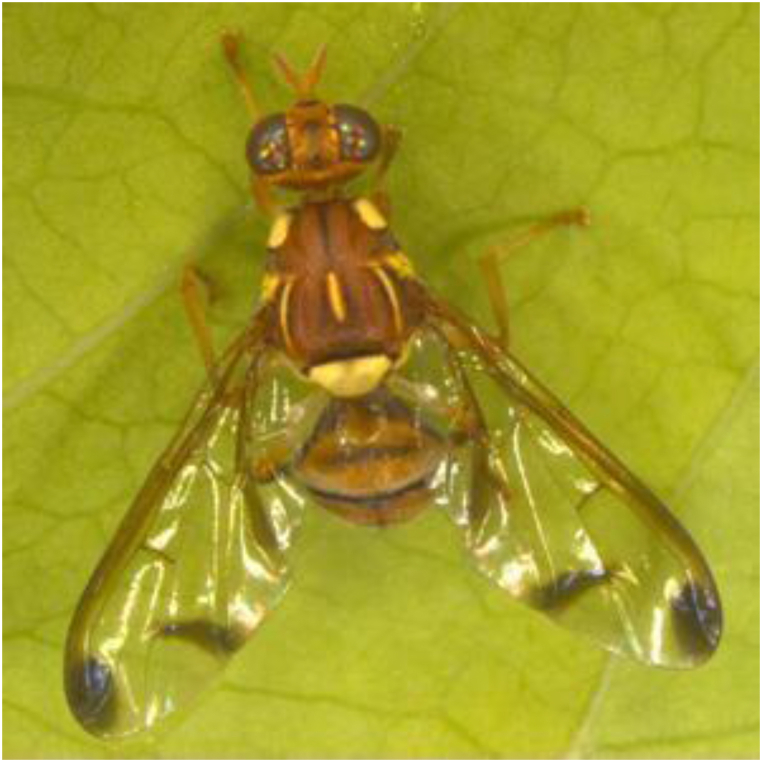
The original image.

**Fig. 6:**
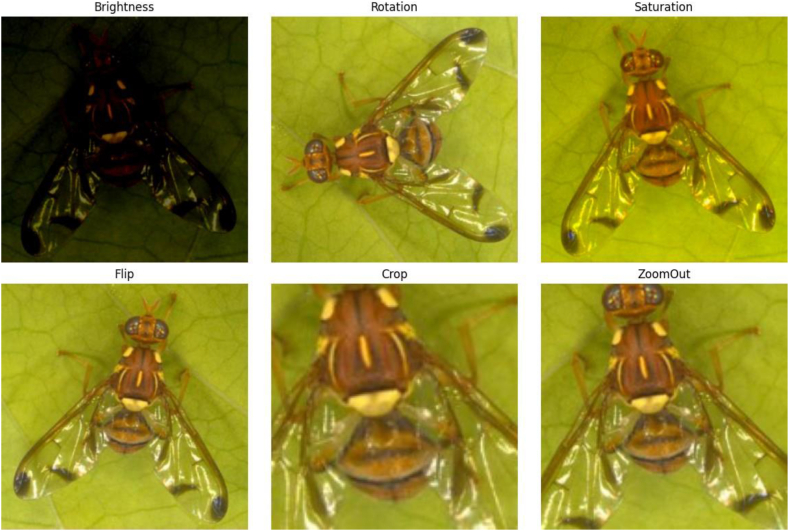
The image after modification when applying image augmentation.

Fig. 5 displays the original image of the Zeugodacus cucurbitae insect; Fig. 6 displays the same image with image augmentation applied, and the modifications made to the original image.

After employing the data augmentation approach, 5894 images were used in total; the data was split into up to 80 % training data and up to 20 % validation data.

### 3.3 Apply deep learning model

Deep learning is characterized by its ability to learn on large datasets, and here CNN was used to learn on a dataset of images of insects that affect crops, especially tomatoes.

#### 3.3.1 CNN model usage

**Fig. 7:**
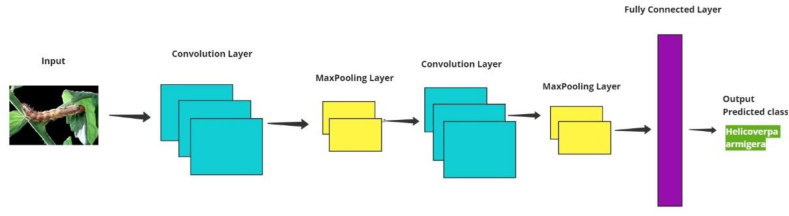
CNN architectures.

**Fig. 8:**
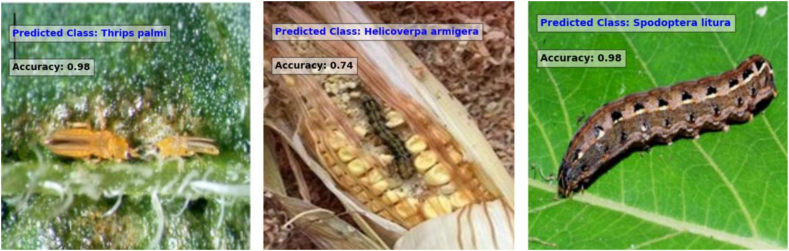
A set of images that were tested on the CNN model with accuracy.

CNN architectures can be found in research, where the use of CNN models has shown remarkable effectiveness in accurate identification [25,26]. A CNN typically has three layers: a convolutional layer, a pooling layer, and a fully connected layer. Fig. 7 Illustrates CNN architectures (see Fig. 8).

## 4 Results and discussions

The findings of the study were documented in this section. Python programming lan-gauge was utilized to implement the code on Google Colab. CNN was utilized, the number of epochs was 40, and the results were registered. Table 3 shows the results.

**Table 3:** Results of the CNN model.

Training accuracy	Validation accuracy	Testing accuracy
90 %	87 %	85 %

Increasing the number of images contributed to increasing accuracy and improving the results, and this is consistent with many studies [23,[27], [28], [29], [30]] that indicated that increasing the amount of data used in training machine learning systems contributes directly.

This study intends to improve augmented reality applications in agriculture, including the early detection of insect pests on tomato crops. A deep-learning CNN model was applied to a set of insect pest images. The CNN model was trained on the image dataset, and image augmentation technologies were used to expand the number of images in the database, with the results noted. Training accuracy achieved 90 %, while test accuracy reached 85 % [25]. This study is consistent with many previous studies, as we have mentioned in this research. The farmer can use the application and benefit from the services of early detection of insect pests that infect tomatoes by integrating the code into an application that supports cell phones that the farmer can use so that the infected leaf is photographed and the disease present is identified.

## CRediT authorship contribution statement

**Moy'awiah Al-Shannaq:** Supervision. **Shahed N. Alkhateeb:** Writing – original draft, Formal analysis, Data curation, Conceptualization. **Mohammad Wedyan:** Writing – review & editing, Writing – original draft.

## Informed consent statement

Not applicable.

## Institutional review board statement

Ethical approval was not necessary for the preparation of this article.

## Data availability statement

The original contributions presented in this study are included in this article, further inquiries can be directed to the corresponding author.

## Code availability

Made available on request.

## Funding

Open Access funding provided partially by 10.13039/501100006418Yarmouk university. No funding applied.

## Declaration of competing interest

The authors declare that they have no known competing financial interests or personal relationships that could have appeared to influence the work reported in this paper.
